# Diagnostic value of the appendicitis inflammatory response (AIR) score. A systematic review and meta-analysis

**DOI:** 10.1186/s13017-025-00582-x

**Published:** 2025-02-08

**Authors:** Roland E. Andersson, Joachim Stark

**Affiliations:** 1 Department of Biomedical and Clinical Sciences, Linköping University, Linköping, Sweden; 2 Futurum Academy for Health and Care, Jönköping County Council, Futurum Läkarprogrammet Hus D2, Länssjukhuset Ryhov, 551 85 Jönköping, Sweden

**Keywords:** Appendicitis, Diagnosis, Scoring system, AIR score, Alvarado score, Risk stratification

## Abstract

**Background:**

Clinical scoring algorithms are cost efficient in patients with suspicion of acute appendicitis. This is a systematic review and meta-analysis of the diagnostic properties of the Appendicitis Inflammatory Response (AIR) score compared with the Alvarado score.

**Methods:**

The PubMed, EMBASE, Web of Science and Google Scholar databases were searched for reports on the diagnostic properties of the AIR score from 2008 to July 18, 2024. A meta-analysis of the receiver operating characteristic (ROC) area and the sensitivity and specificity for all and advanced appendicitis patients was performed. Advanced appendicitis was defined as perforated or gangrenous appendicitis or appendicitis abscess or phlegmon or if described as complicated appendicitis. The risk of bias was estimated via the QUADAS-2 tool. The ROC areas of the AIR score and the Alvarado score were compared.

**Results:**

A total of 26 reports with a total of 15.699 patients were included. The area under the ROC curve for the AIR score was 0.86 (95% CI 0.83–0.88) for all patients with appendicitis and 0.93 (CI 0.91–0.96) for those with advanced appendicitis, which was greater than the corresponding areas for the Alvarado score (0.79, CI 0.76; 0.81) and 0.88, CI 0.82; 0.95), respectively.

At > 4 points, the sensitivity was 0.91 (CI 0.88; 0.94) for all patients with appendicitis and 0.95 (CI 0.94; 0.97) for those with advanced appendicitis. At > 3 points, the sensitivity was 0.95 (0.90; 0.97) for all patients with appendicitis and 0.99 (0.97; 0.99) for those with advanced appendicitis.

At > 8 points, the specificity was 0.98 (0.97; 0.99) for all patients with appendicitis and 0.99 (0.97; 0.99) for those with advanced appendicitis. The included studies had a low risk for bias and low heterogeneity.

**Conclusion:**

The AIR score has a better diagnostic capacity than the Alvarado score does. The AIR score is a safe and efficient basis for risk-stratified management of patients suspected of having appendicitis.

## Background

Acute appendicitis is one of the most important differential diagnoses in patients presenting with acute abdominal pain at emergency departments. Patients with acute abdominal pain and suspicion of appendicitis can present with a wide spectrum of symptoms and signs and severity of the condition. The diagnosis is challenging, and the diagnostic process, especially the use of routine diagnostic imaging, is controversial [1, 2]

Initial management is usually based on the clinical presentation and basic laboratory tests. In the triage, patients with advanced appendicitis need to be recognised and given immediate attention, whereas patients with a presentation suggesting noncomplicated appendicitis, which may be self-limiting, are not an immediate medical emergency [3].

Initial management can be facilitated and made more efficient and safer by the use of clinical scoring systems based on symptoms, signs and simple inflammatory markers, which are part of the routine primary workup. The scoring system can define strata with high, indeterminate and low risk of appendicitis. These three zones can be the basis for an optimal and structured risk stratified pathway. This can involve immediate resuscitation and early diagnostic laparoscopy for patients in the high-risk zone, selective imaging or repeat scoring after observation for the indeterminate zone or early discharge with planned follow-up for the low-risk zone. Such risk-stratified management is safe and more cost efficient than unselective and routine use of imaging [4, 5].

Diagnostic tests are usually analysed from a binary point of view, often trying to define the point with an optimal balance between sensitivity and specificity. Indeterminate results are either not included in the analysis or counted as negative. This can lead to a loss of diagnostic information and bias. The use of three test zones, with one zone with high sensitivity and another with high specificity and an indeterminate zone in between, has been proposed as a solution. This model is closer to clinical reality [6, 7].

Many simple and user-friendly scoring systems have been presented, but few meta-analyses exist [8–11]. This report is a systematic review and meta-analysis of the diagnostic properties of the Appendicitis Inflammatory Response (AIR) score (Table 1), which is the second most cited appendicitis scoring system next to the Alvarado score [12]. The results are analysed and presented from the perspective of three risk zones.

**Table 1:** Appendicitis inflammatory response (AIR) score

Parameters	Points
Vomiting	1
Pain in right iliac fossa	1
Rebound tenderness/abdominal muscle defense	
Slight	1
Moderate	2
Strong	3
Temperature ≥ 38.5 °C	1
Leukocyte concentration	
10–14 × 10^9^/l	1
≥ 15 × 10^9^/l	2
Proportion neutrophils	
70–84%	1
≥ 85%	2
CRP concentration	
10–49 mg/l	1
≥ 50 mg/l	2
Total score	12

The original cut-off point for low risk (≤ 4) has recently been replaced with ≤ 3. A score > 8 points indicates high risk

## Methods

We followed the recommendations of the Preferred Reporting Items for Systematic Reviews and Meta-Analysis (PRISMA) 2020 statement [13].

### Literature search

The PubMed, EMBASE and Web of Science databases were searched for relevant reports, using the search terms (("AIR score" OR "AIRscore" OR "inflammatory response score" OR "appendicitis inflammatory response") AND “appendicitis”). The search started in 2008, the year when the AIR score was published, and stopped on July 18th, 2024. More elaborate search profiles gave the same results as this simple profile. We also included all reports that had a reference to the original publication of the AIR score, according to Google Scholar. Additionally, a direct search was performed on the basis of the authors’ personal knowledge of the literature. After removal of duplicates, the two authors independently performed title and abstract screening as well as full-text screening. Disagreements were discussed and resolved between the authors.

### Inclusion and exclusion criteria

*Inclusion* We included studies reporting the outcome of patients assessed for suspicion of appendicitis in strata according to the AIR-score riskzones, with high (> 8 points) and low risk (≤ 4, ≤ 3 or ≤ 2 points). Some of the included studies reported results from only one of these cut-off points.

**Fig. 1:**
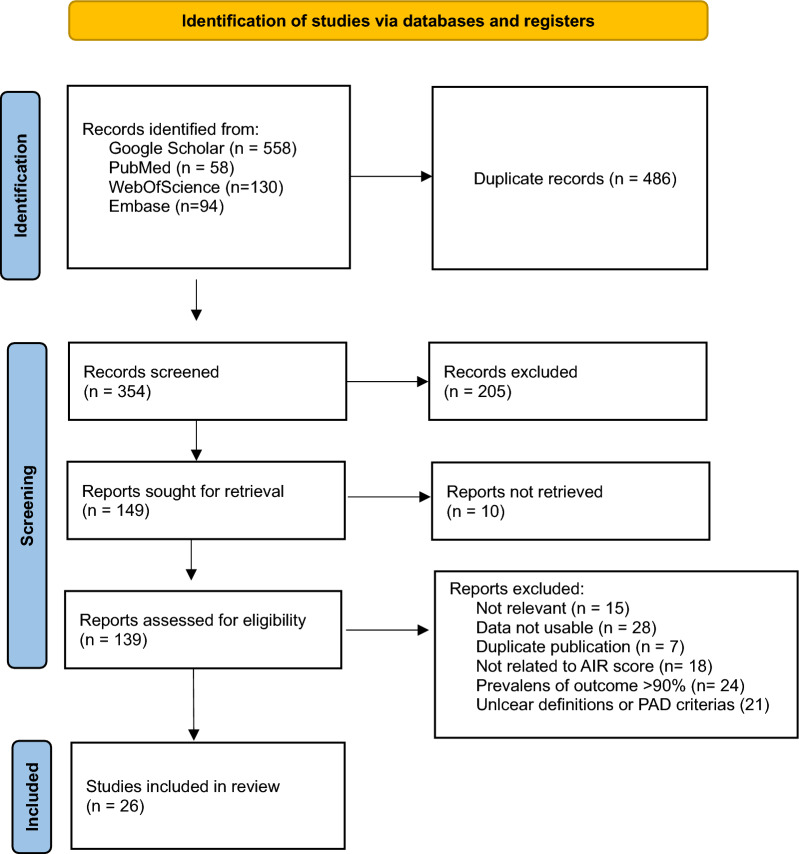
The PRISMA flowchart for the study

*Exclusion* Reports that used very wide criteria for histopathological diagnosis were excluded. The risk scoring strategy is intended for unselected patients with suspicion of appendicitis, typically having a prevalence of appendicitis of approximately 30%. Given that a high prevalence of disease may have an impact on sensitivity and specificity [14], we excluded reports with a greater than 90% prevalence of appendicitis. Case reports, duplicates and reports that did not present results that could be extracted or used for the statistical analyses were excluded. The selection process is shown in the PRISMA flowchart in Fig. 1.

### Data extraction

We extracted relevant information such as study identification (i.e., author and year of publication), study characteristics (i.e., study design, age of the participants, and study period), outcomes (appendicitis and not appendicitis), and use of a reference standard (PAD or follow-up). We noted the number of outcomes according to the low and high diagnostic thresholds of the AIR score (i.e., true/false and positive/negative cases). If not reported, the numbers were derived from the reported statistics. If available, we extracted the corresponding numbers separately for advanced appendicitis. Advanced appendicitis was defined as perforated or gangrenous appendicitis or appendicitis abscess or phlegmon or as complicated appendicitis. The histopathological diagnosis of a normal appendix was made by the authors of the original studies. Two investigators independently assessed the quality of each included study according to the QUADAS-2 tool [15]. Any disagreements were resolved by discussion between the investigators.

### Statistical analysis

For each report, the results were extracted as the number of patients in four strata—true/false and positive/negative. This was performed for all patients with appendicitis and, if available, for those with advanced appendicitis. If available, data were extracted for cut-off points > 3, > 4 and > 8. One study reported results with a cut-off point > 2 only. The performance at ruling out appendicitis in the low-risk strata was tested from the sensitivity in the low- versus the combined intermediate- and high-risk strata. The performance in ruling out appendicitis in the high-risk strata was tested in terms of specificity in the high-risk strata versus the combined intermediate- and low-risk strata. For each study, we calculated the sensitivity and specificity for the low and high cut-off points separately and estimated the corresponding pooled results in a meta-analysis. The ROC area for the AIR score and, if available from the Alvarado score, was extracted from each report and compared statistically from the standardised mean difference. Separate analyses were performed for all patients with appendicitis and those with advanced appendicitis. Heterogeneity between studies was assessed by the I2 index. We used STATA software (Version 17.0, StataCorp, College Station, TX) for all the statistical analyses. The meta-analysis was performed via the “metadta” and “metan” commands.

## Results

The literature search yielded 58 hits in PubMed, 94 hits in EMBASE and 130 hits in Web of Science. We also included 558 references to the original publication of the AIR score listed in Google Scholar. After exclusion of duplicates, reports unrelated to the AIR score and reports from which we could not extract any useful data, 139 potential references were retrieved for assessment of eligibility. After the full texts were assessed, another 109 reports were excluded, leaving 26 reports for the final analysis, with a total of 15.699 included patients. One study reported separate results for males and females, which explains 27 sets of data (Fig. 1, Table 2).

**Table 2:** Characteristics of the studies included in the meta-analysis

References	Timing	Inclusion criteria	Number of patients		Prevalence appendicitis (%)	Proportion perforated (%)	Country
			Total	Operated			
[16]	Pro	Suspected acute appendicitis	229	129	33.2	39.5	Sweden
[17]	Pro	Suspected acute appendicitis	428	206	41.4	38.4	Sweden
[18]	Pro	Suspected acute appendicitis, age > 5y	3878	1598	39.8	46.9	Sweden
[19]	Retro	Acute appendectomy, pregnant	53	53	75.5	?	Turkey
[20]	Pro	Suspected acute appendicitis, age 16–45	5345	1957	27.4	?	UK
[21]	Pro	Suspected acute appendicitis, age 15–70, unclear sampling	89	89	78.7	?	Mexico
[22]	Pro	Acute appendectomy, age > 12	107	107	84.1	?	India
[23]	Pro	Suspected acute appendicitis	941	435	36.8	29.5	Netherlands
[24]	Pro	Suspected acute appendicitis, age > 16, unclear sampling	100	76	68.0	?	Egypt
[25]	Retro	Acute appendectomy	424	424	74.3	30.2	N Zealand
[26]	Pro	Suspected acute appendicitis, age > 16	245	198	71.8	46.6	Malaysia
[27]	Pro	Suspected acute appendicitis	300	242	38.7	24.1	India
[28, 29]	Pro	Suspected acute appendicitis, age < 15	318	150	47.5	50.3	Sweden
[30]	Retro	Acute appendectomy	73	73	80.8	13.6	UK
[31]	Pro	Acute appendectomy	130	130	89.2	11.2	India
[32]	Retro	Acute appendectomy	201	201	82.6	41.6	Ireland
[33]	Retro	Suspected acute appendicitis, pregnant, MRI	255	29	10.2	11.5	Israel
[34]	Pro	Acute appendectomy, age > 18	328	328	86.6	?	Iraq
[35]	Pro	Suspected acute appendicitis	182	74	36.8	23.9	Ireland
[36]	Retro	Suspected acute appendicitis, pregnant, nested case‒control	386	154	30.3	35.0	Sweden
[37]	Pro	Acute appendectomy, stratified random	100	100	89.0	?	India
[38]	Pro	Suspected acute appendicitis, age > 18	218	114	49.1	?	Turkey
[39]	?	Acute appendectomy, age > 18	120	120	85.8	22.3	Iran
[40]	Pro	Suspected acute appendicitis, age > 15	725	421	47.3	?	Finland
[41]	Pro	Suspected acute appendicitis	464	216	28.4	38.6	UK
[42]	Pro	Acute appendectomy	60	60	88.3	?	India

### Characteristics of the included studies

The final selected reports come from many countries and settings (Table 2) [16–42]. The majority of these studies are prospective and include consecutive patients. Approximately half of the reports are based on patients assessed for suspicion of appendicitis, and the remaining include patients operated on for suspicion of appendicitis. The median prevalence of appendicitis was 56.6% (range 12.5–89.2%).

Most reports include patients of both sexes and all ages or adults. One study involving children reported results in which > 4 was used as the low cut-off point [28] and > 3 was used as the low cut-off point in a complementary report [29]. Three reports included only pregnant women [19, 33, 36].

For the operated patients, the final diagnosis was based on histopathology in all the reports (Table 3). For the unoperated patients, the diagnosis was based on radiologic examination in two studies and results after between 2 weeks and 6 months of follow-up in 13 studies (Table 3). The criteria used for the histopathological diagnosis of appendicitis were transmural inflammation in eight reports, neutrophil invasion to the muscularis propria in six reports and various other criteria in three reports (Table 3). Reports accepting the presence of neutrophils in the lumen or invasion limited to the mucosa or lymphoid hyperplasia as criteria were excluded. The remaining reports did not specify the histopathological criteria used.

**Table 3:** Diagnostic criteria used

Reference	Diag	Follow up	Minimum histopathology criteria	Advanced app definitions
[16]	HP	1 month chart	Transmural inflammation	30 gangrenous or perforated
[17]	HP	6 month chart	Transmural inflammation	68 gangrenous or perforated
[18]	HP	30d chart	Transmural neutrophil infiltration	724 gangrenous or perforated or abscess
[19]	HP	N/A	Unclear	Unclear
[20]	HP	30d chart	Unclear	Unclear number gangrenous or perforated
[21]	HP	N/A	Unclear	Unclear
[22]	HP	N/A	Unclear	Unclear
[23]	HP	"Routine"	Muscularis propria neutrophil infiltration	92 gangrenous/perforated + 10 abscess
[24]	HP	2 weeks	Transmural neutrophil infiltration	Unclear
[25]	HP	N/A	Muscularis propria neutrophil infiltration	57 gangrenous + 38 perforated
[26]	HP/CT	Radiology or 3 month chart	Unclear	78 perforated + 4 abscess
[27]	HP	"Routine"	Muscularis propria neutrophil infiltration	28 unclear
[28, 29]	HP	1 month chart	Muscularis propria neutrophil infiltration	76 gangrenous/perforated/abscess
[30]	HP	N/A	Unclear	5 gangrenous + 3 perforated
[31]	HP	N/A	Muscularis propria neutrophil infiltration	13 perforated
[32]	HP	N/A	Unclear	17 gangrenous + 52 perforated
[33]	HP/MRI	Unclear	Unclear	2 abscess + 1 periapp phlegmon
[34]	HP	N/A	Unclear	Unclear
[35]	HP	Unclear	Transmural inflammation	16 gangrenous or perforated
[36]	HP	30d chart	Transmural inflammatory cell infiltration	41 gangrenous/perforated/abscess
[37]	HP	N/A	Unclear	Unclear
[38]	HP	Radiology and 2 weeks	Unclear	Unclear
[39]	HP	N/A	Unclear	23 perforated
[40]	HP	2 weeks chart	Transmural neutrophil infiltration	Unclear number perforated or abscess
[41]	HP	30 days	Transmural neutrophil infiltration	51 gangrenous/perforated/abscess
[42]	HP	N/A	Unclear	Unclear

The area under the ROC curve (AUC) for the AIR score was reported in 23 studies for all patients with appendicitis (one study reporting separate results for men and women) and in 7 studies for patients with advanced appendicitis. Some 22 studies reported ROC areas for both the AIR and the Alvarado score for all patients with appendicitis and three for those with advanced appendicitis.

The number of true/false and positive/negative patients related to the low (> 2, > 3 or > 4 points) and high (> 8 points) AIR score cut-off points were extracted from the reports, and the corresponding sensitivity and specificity were calculated. Separate analyses were performed for all patients and for those with advanced appendicitis. Three studies reported results for both > 3 and > 4 points as the low cut-off points [18, 25, 28, 29]. One study reported results for men and women separately, using two different cut-off points [20].

### Quality assessment

The included studies were generally of high quality. In the QUADAS-2 assessment, 13 of the 26 studies had a high risk of bias and concern regarding applicability in the domain of patient selection due to the exclusion of nonoperated and/or nonpregnant patients (Table 4). Some 16 studies had a high risk of bias and concern regarding applicability in the domain flow and timing, mainly because different reference tests were applied (histopathology of operated patients vs follow-up of nonoperated patients). Fourteen studies were unclear with respect to both risk of bias and concern regarding applicability in the domain reference standard, mainly because they were unclear or did not specify histopathological criteria.

**Table 4:** QUADAS-2 adjudgments of included studies

Study	Risk of bias				Applicability		
	P	I	R	FT	P	I	R
[16]	✓	✓	✓	X	✓	✓	✓
[17]	✓	✓	✓	X	✓	✓	✓
[18]	✓	✓	✓	X	✓	✓	✓
[19]	X	?	?	✓	X	✓	?
[20]	✓	X	?	X	✓	✓	?
[21]	X	✓	?	X	X	✓	?
[22]	X	✓	?	✓	X	✓	?
[23]	✓	✓	✓	X	✓	✓	✓
[24]	?	✓	✓	X	✓	✓	✓
[25]	X	?	✓	✓	X	✓	✓
[26]	✓	✓	?	X	✓	✓	?
[27]	✓	✓	✓	X	✓	✓	✓
[28, 29]	✓	✓	✓	X	✓	✓	✓
[30]	X	?	?	✓	X	✓	?
[31]	X	✓	✓	✓	X	✓	✓
[32]	X	✓	?	✓	X	✓	?
[33]	X	?	?	X	X	✓	?
[34]	X	✓	?	✓	X	✓	?
[35]	✓	✓	?	X	✓	✓	?
[36]	X	?	✓	X	X	✓	✓
[37]	X	✓	?	✓	X	✓	?
[38]	✓	✓	?	X	✓	✓	?
[39]	X	?	?	✓	X	✓	?
[40]	✓	✓	✓	X	✓	✓	✓
[41]	✓	✓	✓	X	✓	✓	✓
[42]	X	✓	?	✓	X	✓	?
N low:	12	19	12	10	13	26	12
N unclear:	1	6	14	0	0	0	14
N high:	13	1	0	16	13	0	0

*P* patient selection, *I* index test, *R* reference standard, *FT* flow and timing

✓ indicates low risk; X indicates high risk; ?? indicates unclear risk

### Diagnostic accuracy

#### Overall diagnostic accuracy

A classifier's performance can be described by the area under the ROC curve. For the AIR score, the pooled ROC area for all cases of appendicitis was 0.86 (95% CI 0.83; 0.88) in 23 studies and 0.93 (CI 0.91; 0.96) for advanced appendicitis in 7 studies.

**Fig. 2:**
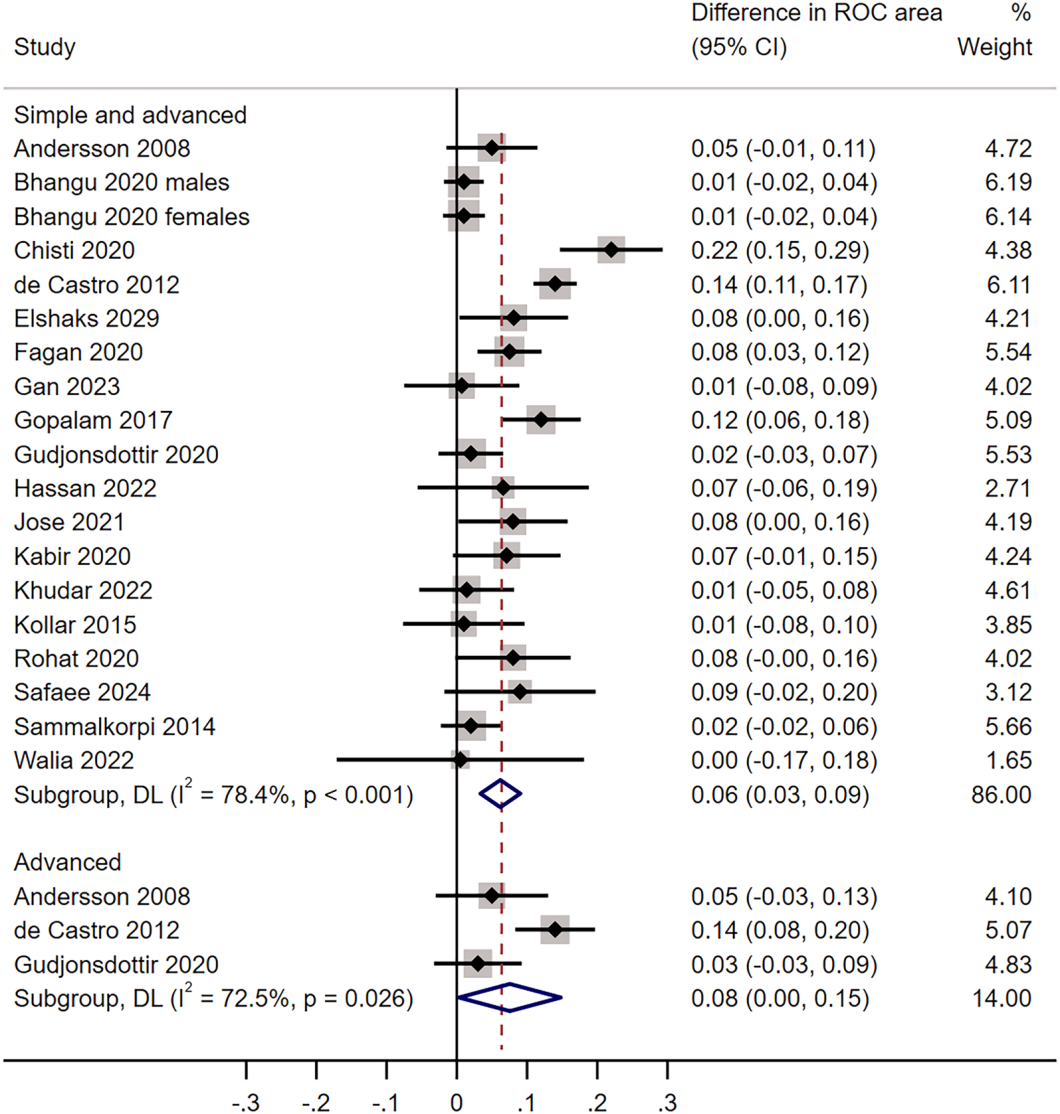
Standardised mean difference for the paired ROC areas. The pooled ROC area for all patients with appendicitis was 0.85 (CI 0.82; 0.88) for the AIR score and 0.79 (CI 0.76; 0.81) for the Alvarado score, with a difference of 0.06 (CI 0.03; 0.09, *p* < 0.001). The corresponding result for advanced appendicitis was 0.96 (CI 0.94; 0.98) for the AIR score compared with 0.88 (CI 0.82; 0.95) for the Alvarado score in three reports, a difference of 0.08 (CI 0.00;0.15), *p* = 0.03. Gudjonsdottir [28] included only children. CI is Confidence Interval

The AIR score and the Alvarado score were both reported in 19 studies. For these paired reports, the pooled ROC area for all cases of appendicitis was significantly larger for the AIR score (0.85, CI 0.82; 0.89) than for the Alvarado score (0.79, CI 0.76; 0.81, *p* < 0.001). For advanced appendicitis, the pooled ROC area was 0.96 (CI 0.94; 0.98) for the AIR score compared with 0.88 (CI 0.82; 0.95) for the Alvarado score in three reports (*p* < 0.001). An analysis of the weighted mean difference in the ROC area for the AIR and the Alvarado score revealed a significantly larger ROC area for the AIR score (Fig. 2).

#### Diagnostic accuracy at the low cut-off point

**Fig. 3:**
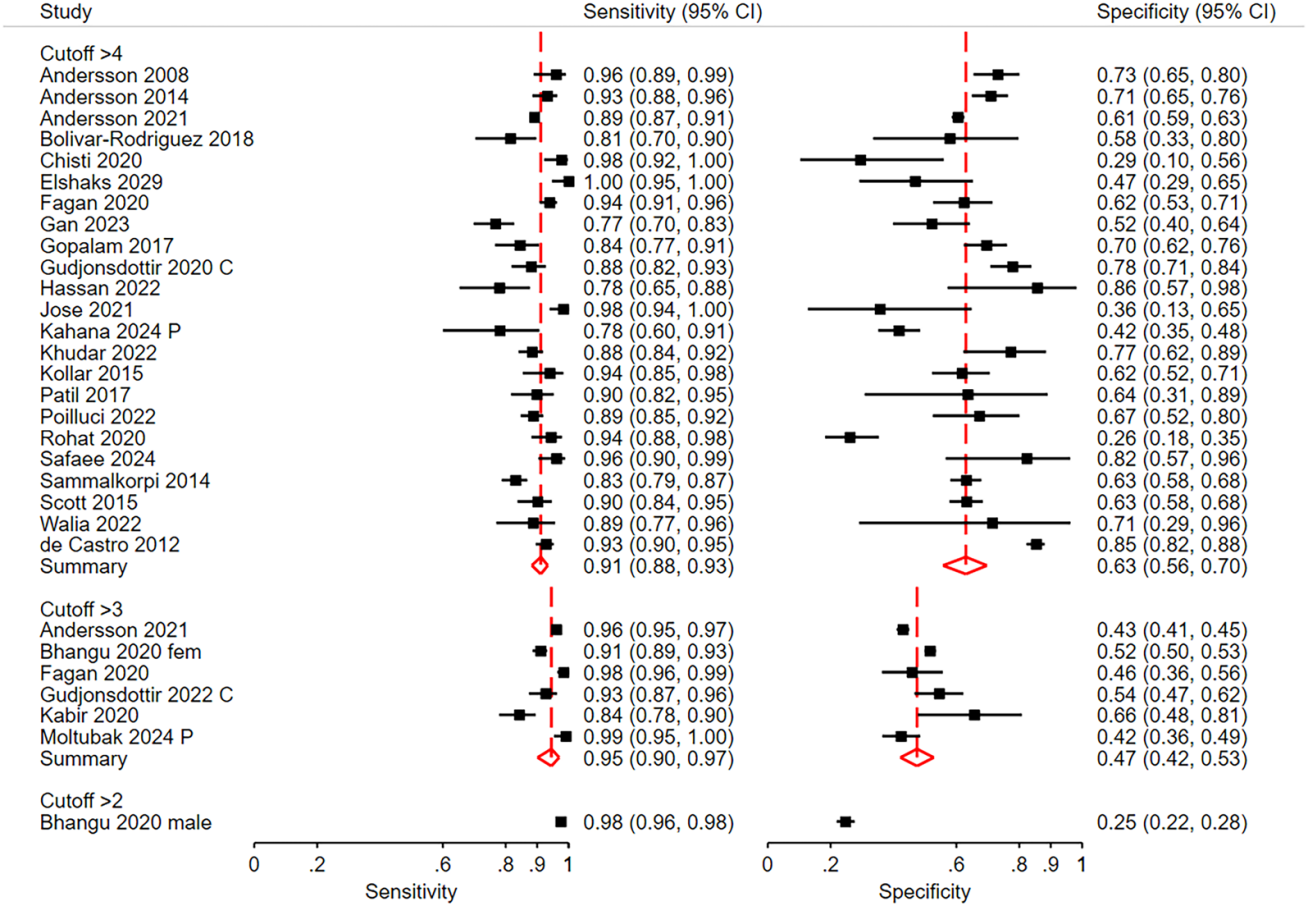
Sensitivity and specificity for all patients with appendicitis at the low cut-off point. The aim at this cut-off is high sensitivity. Gudjonsdottir [28] C included only children and Moltubak [36] P included only pregnant women. Bhanghu [20] presented results for females and males separately. CI is Confidence Interval

**Fig. 4:**
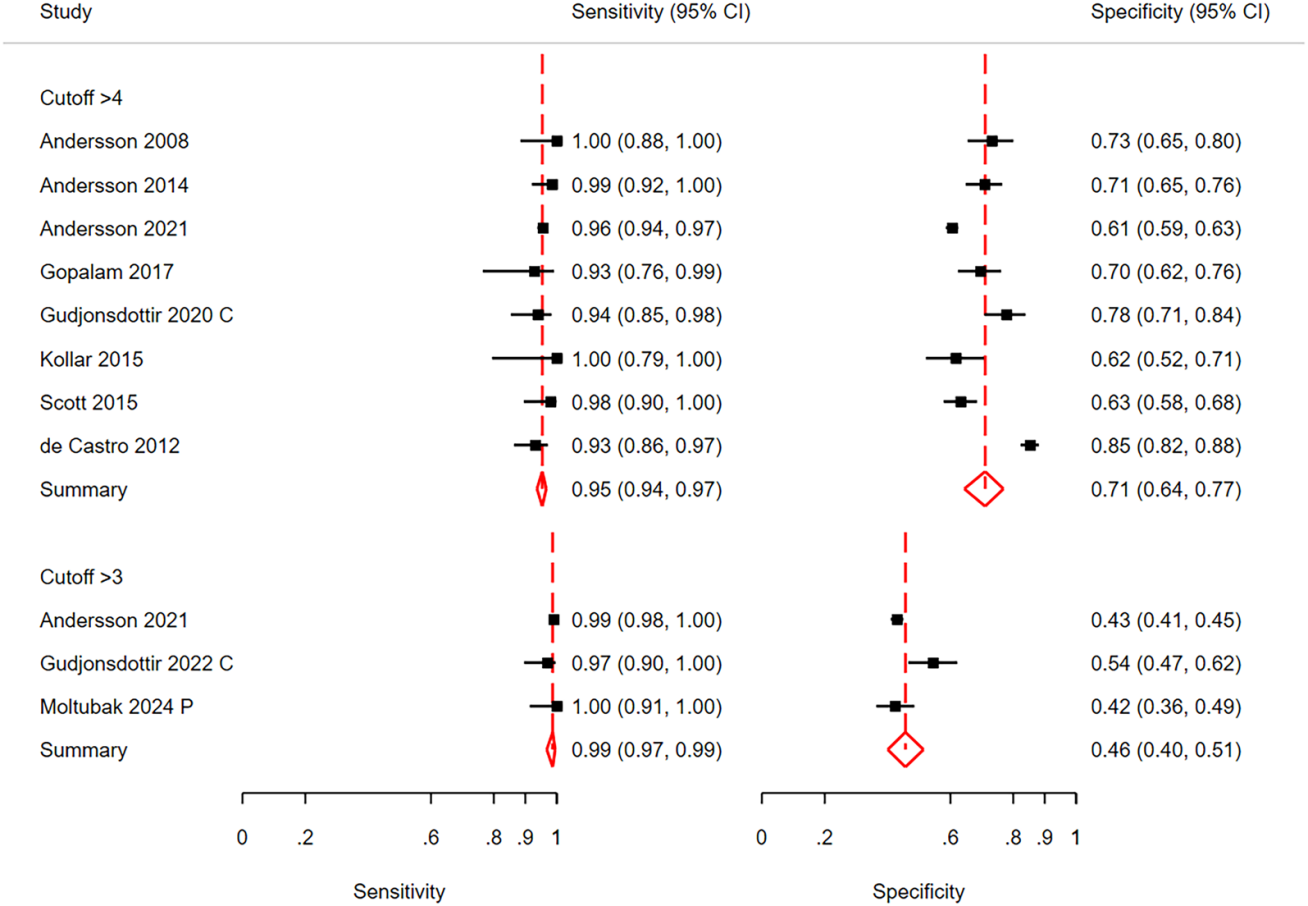
Sensitivity and specificity for advanced appendicitis at the low cut-off point. Gudjonsdottir [28] C included only children and Moltubak [36] P included only pregnant women. CI is Confidence Interval

At the low cut-off point, the aim of the AIR score is to obtain high sensitivity, especially for advanced appendicitis. A score > 4 points had a pooled sensitivity of 0.91 (CI 0.88; 0.94) for all patients with appendicitis (Fig. 3) and a pooled sensitivity of 0.95 (0.94; 0.97) for patients with advanced appendicitis vs those without appendicitis (Fig. 4). At a cut-off point of > 3, the pooled sensitivity was 0.95 (0.90; 0.97) for all patients with appendicitis and 0.99 (0.97; 0.99) for those with advanced appendicitis. The corresponding pooled specificities were 0.63 (0.55; 0.70) and 0.71 (0.64; 0.77) at a cut-off > 4 and 0.47 (0.42; 0.53) and 0.46 (0.40; 0.51) at a cut-off > 3, respectively.

#### Diagnostic accuracy at the high cut-off point

**Fig. 5:**
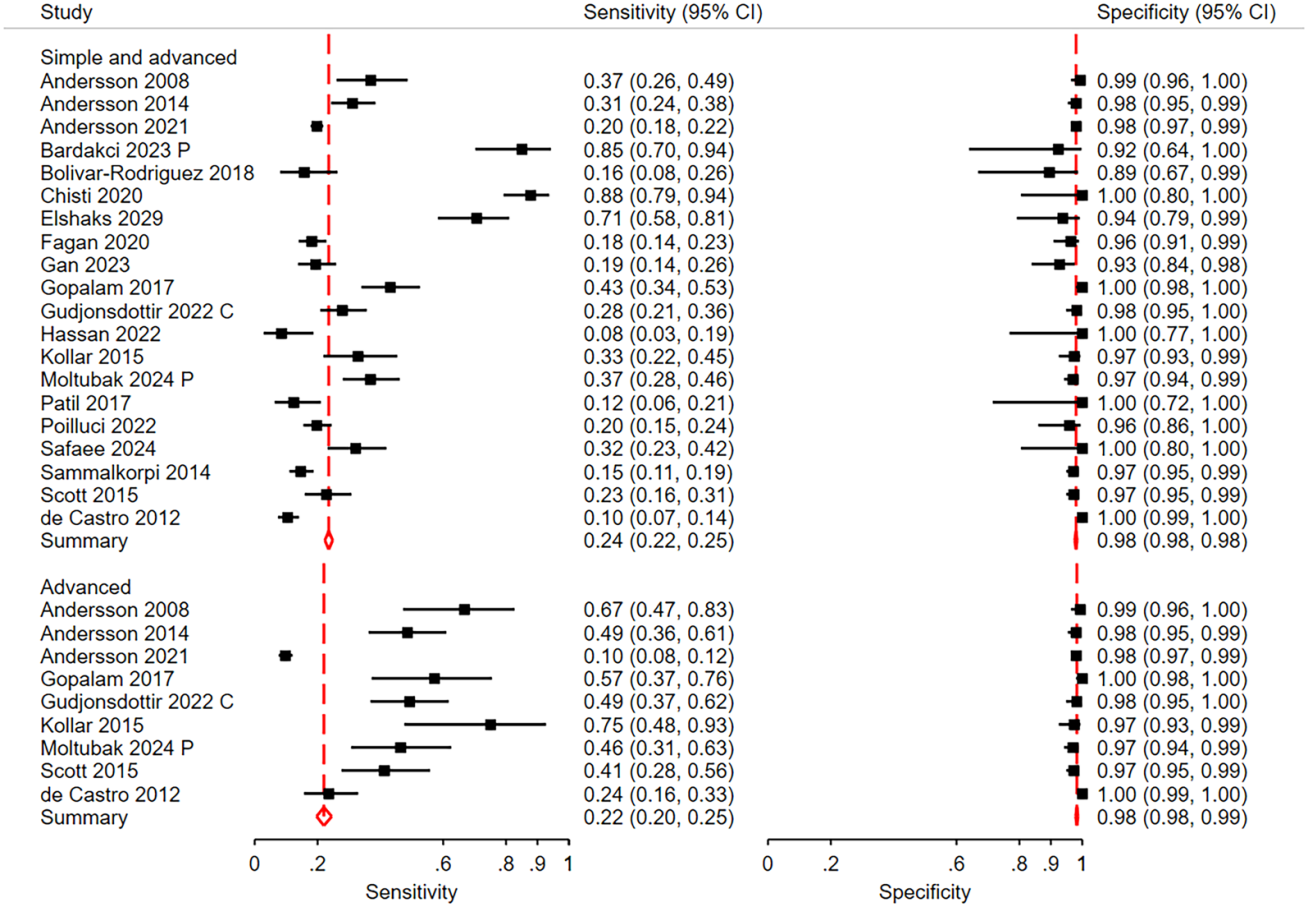
Sensitivity and specificity for all patients and for those with advanced appendicitis at the high cut-off point (> 8 points). Gudjonsdottir [28] C included only children and Moltubak [36] P included only pregnant women. CI is Confidence Interval

At the high cut-off point, the aim is to identify patients with appendicitis, especially advanced appendicitis, with high specificity. At a cut-off point > 8, the pooled specificity of the AIR score was 0.98 (0.97; 0.99) for all patients with appendicitis and 0.99 (0.97; 0.99) for those with advanced appendicitis (Fig. 5). The corresponding pooled sensitivities were 0.30 (0.21; 0.42) and 0.43 (0.29; 0.59), respectively.

## Discussion

The traditional understanding of appendicitis as a single entity, where inflammation progresses and eventually leads to perforation if untreated, is currently questioned. Accumulating evidence suggests that simple and advanced appendicitis represent two different entities and that simple appendicitis may resolve without treatment [3, 43–45]. As a consequence, management aimed at early diagnosis and immediate surgical treatment to prevent perforation is now replaced by more diversified management. The main aim is early detection and treatment of patients with advanced appendicitis from the large majority of patients suspected of having appendicitis. If advanced appendicitis is unlikely, a second evaluation after a short period of observation is safe and cost efficient compared with routine diagnostic imaging [5].

The use of an algorithm based on a clinical scoring system can make this management more objective and efficient. The AIR score is the second most cited appendicitis risk score, next to the Alvarado score [12]. The AIR score is based on four inflammatory variables and two signs of peritoneal irritation (Table 1). It was designed with a special focus on identifying patients with advanced appendicitis [16].

**Fig. 6:**
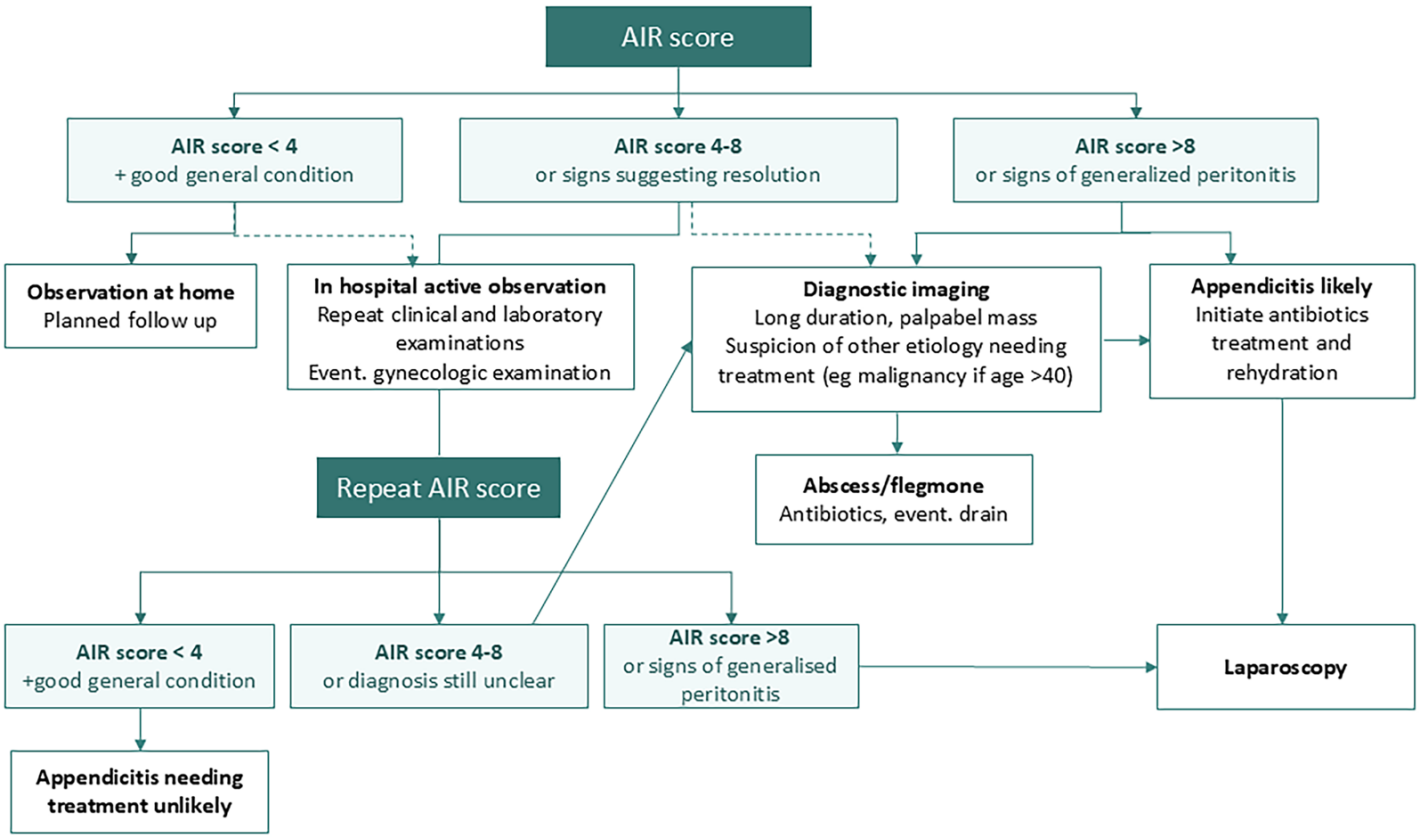
The AIR score algorithm

The AIR score-based algorithm uses two cut-offs to define three groups of patients with high, medium and low risk of appendicitis (Fig. 6). The aim of the high-risk cut-off is to identify patients with appendicitis with high specificity. In this meta-analysis, approximately 25% of all patients with appendicitis were assigned to the high-risk group, with a specificity of 0.98 and a prevalence of appendicitis of 91%. These patients need urgent surgical evaluation and probable abdominal diagnostic exploration. At such a high prevalence, imaging cannot rule out appendicitis but will only yield a high proportion of false negative results [46]. However, imaging may be indicated for differential diagnosis if other inflammatory conditions need to be ruled out.

Conversely, the aim of the low cut-off is to define a group of patients with a very low probability of advanced appendicitis, where reexamination after observation can be motivated [5]. In this meta-analysis, the low cut-off had a sensitivity for advanced appendicitis of 0.95 at a score > 4 and 0.99 at a score > 3. With an AIR score ≤ 4, approximately 40% of all the patients were assigned to the low-risk group, with a prevalence of advanced appendicitis of 1%. At an AIR score ≤ 3, the proportion of patients with advanced appendicitis was only 0.3%. At such a low prevalence, imaging would yield many false positive results and an increased risk for negative appendectomies [46]. A planned reexamination after a short period of expectant management is a safe alternative in this group of patients [5].

### Study limitations

Although histopathology is the gold standard for the diagnosis of appendicitis, the final diagnosis is strongly dependent on the criteria for the findings on histopathology [47]. Some studies reported that the diagnosis was based on histopathological examination but did not declare the criteria used. Three studies were excluded because they had too wide criteria, such as lymhoid hyperplasia or the presence of neutrophils in the mucosa or even in the appendix lumen.

The inclusion of patients varied from patients with abdominal pain and suspicion of appendicitis to patients who underwent surgery for suspicion of appendicitis. This is reflected in the strong variation in the prevalence of appendicitis. As the estimates of the diagnostic values are influenced by the prevalence [14], we excluded all reports with a prevalence of appendicitis over 90%. However, the high median prevalence is still higher than that in an emergency department where the AIR score should be used for risk stratification of unsorted patients with abdominal pain and suspicion of appendicitis, which typically has a prevalence of approximately 30%.

## Conclusion

This meta-analysis revealed that the AIR score has a significantly better diagnostic performance than the Alvarado score does, as shown by the pooled ROC area. The diagnostic properties of the AIR score at the low and high cut-off points suggest that it is a safe and suitable basis for risk-stratified management of patients suspected of having appendicitis.

## Data Availability

No datasets were generated or analysed during the current study.
